# Supplementary material for: Microbial Community Structure Driven by a Volcanic Gradient in Glaciers of the Antarctic Archipelago South Shetland
Source: Microorganisms. 2021 Feb 14;9(2):392. doi: 10.3390/microorganisms9020392 (PMC7917679; doi:10.3390/microorganisms9020392)

Supplementary Materials for:

Microbial community structure driven by a volcanic gradient in the Antarctic Archipelago South Shetland

*Eva García-Lopez, Sandra Serrano, Miguel Angel Calvo, Sonia Peña, Silvia Sanchez-Casanova Laura García-Descalzo and Cristina Cid**

* Corresponding Author: cidsc@inta.es

This file includes:

I. Supplementary Table: S1-S3

II. Supplementary Figures: S1-S4

I. Supplementary Table

Table S1. Elemental concentrations in melt water.

	Deception		Livingston		Greenwich		King George	
	ROJ	MAC	JOH	HUR	QUI	TRA	ECO	MPI
**Ag**	BD[a]	0.002 (0.001)	0.021 (0.001)	0.088 (0.000)	0.121 (0.001)	0.084 (0.002)	0.079 (0.003)	0.065 (0.003)
**Al**	0.627 (0.120)	0.258 (0.004)	BD	BD	BD	BD	BD	BD
**As**	0.226 (0.003)	0.065 (0.004)	BD	BD	BD	0.321 (0.004)	0.338 (0.005)	0.464 (0.022)
**B**	13.499 (3.225)	2.146 (1.700)	BD	3.300 (0.630)	5.528 (0.336)	8.666 (1.336)	12.345 (2.669)	16.857 (2.700)
**Ba**	5.060 (0.635)	0.474 (0.021)	0.633 (0.001)	BD	BD	BD	2.042 (0.021)	2.054 (0.033)

**Be**	0.010 (0.001)	0.013 (0.032)	0.005 (0.001)	BD	0.320 (0.004)	BD	0.011 (0.004)	0.088 (0.001)
**Bi**	BD	BD	0.124 (0.007)	0.211 (0.006)	1.441 (0.004)	0.369 (0.001)	0.202 (0.021)	0.008 (0.021)
**Br**	7.740 (2.367)	21.597 (7.254)	10.741 (1.254)	9.214 (2.369)	50.247 (5.375)	393.390 (45.778)	465.088 (46.335)	481.294 (10.478)
**C**	4593.276 (214.778)	4000.280 (879.225)	3373.132 (214.478)	1019.992 (52.144)	1094.543 (50.214)	2147.354 (588.214)	3372.365 (244.321)	3047.831 (354.877)
**Ca**	1083.320 (20.789)	547.369 (74.222)	BD	99.654 (14.022)	BD	BD	79.052 (11.225)	BD
**Cd**	3.932 (0.077)	7.116 (1.224)	BD	BD	BD	BD	BD	0.066 (0.004)
**Cl**	47406.605 (1.247)	BD	BD	BD	BD	1.478 (0.240)	113051.469 (1.225)	118931.314 (14.225)
**Co**	BD	0.073 (0.003)	0.018 (0.002)	BD	0.045 (0.004)	0.030 (0.001)	0.020 (0.000)	0.012 (0.001)
**Cr**	0.345 (0.002)	0.211 (0.011)	BD	BD	BD	BD	BD	0.093 (0.002)
**Cu**	11.355 (0.522)	11.869 (0.744)	11.812 (0.211)	10.258 (1.225)	8.721 (1.336)	9.354 (2.547)	11.929 (3.214)	13.348 (1.224)
**Fe**	20.967 (3.254)	15.369 (4.217)	3.560 (0.544)	10.369 (1.224)	BD	1.330 (0.333)	BD	BD
**Ga**	BD	0.004 (0.001)	BD	0.010 (0.002)	0.033 (0.001)	0.014 (0.002)	BD	0.004 (0.001)
**Ge**	BD	0.003 (0.000)	BD	BD	BD	BD	BD	BD
**K**	808.828 (50.226)	310.048 (71.366)	137.269 (33.666)	125.698 (21.547)	118.292 (32.144)	54.369 (3.214)	1833.946 (25.221)	1680.072 (88.221)
**Li**	3.327 (1.224)	0.095 (0.012)	BD	BD	0.214 (0.005)	0.300 (0.012)	0.308 (0.033)	0.628 (0.022)
**Mg**	5400.494 (33.211)	3955.000 (65.214)	28.408 (2.369)	14.258 (3.655)	207.091 (5.698)	1354.159 (210.366)	110.659 (23.364)	2350.691 (562.314)
**Mn**	1.563 (0.211)	3.317 (0.214)	1.160 (0.321)	1.159 (0.333)	1.158 (0.321)	4.369 (0.986)	6.504 (1.123)	7.355 (1.225)
**Mo**	0.051 (0.002)	0.014 (0.003)	0.154 (0.021)	0.254 (0.021)	0.583 (0.049)	0.458 (0.055)	0.328 (0.023)	0.205 (0.008)
**Na**	1173.431	2269.103	765.198	700.154	665.643	874.321	20269.703	18269.703

	(236.147)	(6.366)	(63.321)	(50.654)	(20.354)	(22.685)	(2147.369)	(3.254)
**Ni**	35.357 (2.369)	34.792 (5.487)	26.801 (5.698)	10.369 (6.321)	0.231 (0.054)	11.254 (1.566)	26.716 (0.251)	28.899 (0.999)
**P**	1.878 (0.014)	8.576 (1.123)	14.981 (3.214)	BD	37.028 (1.654)	BD	124.052 (54.214)	125.369 (12.365)
**Pb**	0.050 (0.002)	0.074 (0.005)	0.009 (0.002)	BD	BD	0.002 (0.001)	BD	BD
**Rb**	0.260 (0.003)	0.070 (0.006)	0.059 (0.006)	0.084 (0.008)	0.102 (0.044)	0.254 (0.055)	0.479 (0.021)	0.544 (0.005)
**S**	16258.354 (1478.654)	14509.753 (1456.325)	BD	BD	BD	12698.000 (541.326)	11748.304 (232.666)	6733.097 (9.321)
**Sc**	BD	0.015 (0.003)	BD	0.123 (0.014)	0.288 (0.044)	0.111 (0.022)	BD	0.022 (0.002)
**Se**	BD	BD	BD	BD	1.525 (0.012)	1.200 (0.055)	1.012 (0.211)	BD
**Si**	39.693 (1.254)	30.654 (1.224)	BD	BD	BD	BD	BD	BD
**Sr**	15.342 (1.369)	1.044 (0.006)	1.073 (0.006)	2.365 (0.547)	3.058 (0.598)	10.258 (1.365)	24.591 (2.323)	26.740 (6.333)
**Ti**	6.155 (1.254)	1.389 (0.006)	BD	BD	BD	BD	BD	7.084 (0.987)
**V**	0.780 (0.005)	0.100 (0.002)	0.040 (0.001)	0.090 (0.008)	0.115 (0.025)	0.222 (0.021)	0.400 (0.020)	0.569 (0.005)
**Y**	BD	BD	BD	BD	0.060 (0.009)	0.010 (0.002)	0.016 (0.002)	0.005 (0.001)
**Zn**	62.056 (10.369)	16.980 (2.156)	10.932 (0.009)	12.547 (1.354)	15.201 (1987.321)	20.147 (5.365)	29.659 (2.366)	35.105 (3.221)
**Zr**	BD	BD	0.009 (0.003)	0.054 (0.005)	0.062 (0.055)	0.024 (0.003)	0.005 (0.001)	BD

Values represent averages and s.d. (in parentheses) calculated from three sampling points from each study site; Elemental concentrations are in ppb; [a]BD: below detection.

Table S2. Analysis of bacterial 16S retrieved from ice samples. OTU level aggregate counts of 3 sampling replicates.

File: Table S2.xlsx

Table S3. Analysis of eukaryotic 18S rRNA retrieved from ice samples. OTU level aggregate counts of 3 sampling replicates.

File: Table S3.xlsx

Table S4. Number of sequences belonging to the most abundant OTU in each sampling point

Glacier		ROJ	MAC	JOH	HUR	QUI	TRA	ECO	MPI
**Bacteria**	**OTU**[a]	*Sulfobacillus yellowstonensis*	*Polaromonas*	*Polaromonas*	*Pedobacter*	*Flavobacterium*	*Pedobacter*	*Clostridium frigoris*	*Sphingomonas*
	**No. sequences**[b]	6548	17501	69004	19897	13654	3332	38406	4348
**Eukarya**	**OTU**[a]	*Thalassiosira antarctica*	*Hydrurus foetidus*	Glissomonadida	*Bodomorpha* sp.	*Cocconeis stauroneiformis*	*Urospora neglecta*	*Sporobolomyces roseus*	*Hyphozyma variabilis*
	**No. sequences**[b]	22915	64913	76908	49696	69106	80437	36098	*33857*

[a]Most abundant OTU in each glacier
[b]Number of total sequences from the most abundant OTU in each glacier

II. Supplementary Figures

Figure S1. - Rarefaction curves determined for 16S rRNA and 18S rRNA gene clones. Rarefaction curves indicate the observed OTUs at a genetic distance of 3%. (A) Bacteria. (B) Eukarya.

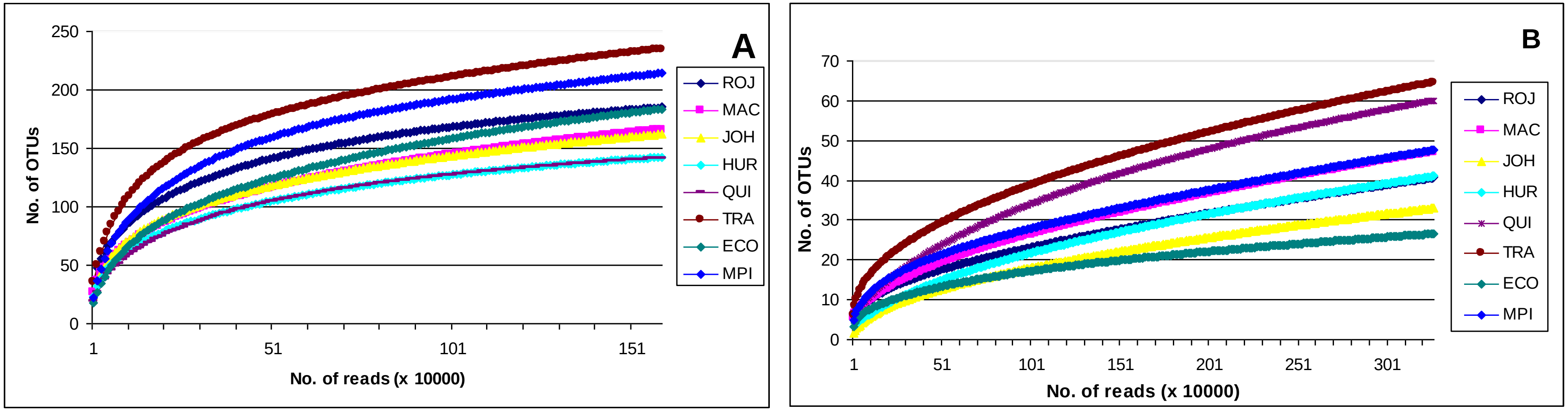

Figure S2. - Number of bacterial sequences and microeukaryote sequences along the volcanic gradient. Graphic representation of the number of 16S rRNA and 18S rRNA sequences and their corresponding trend lines.

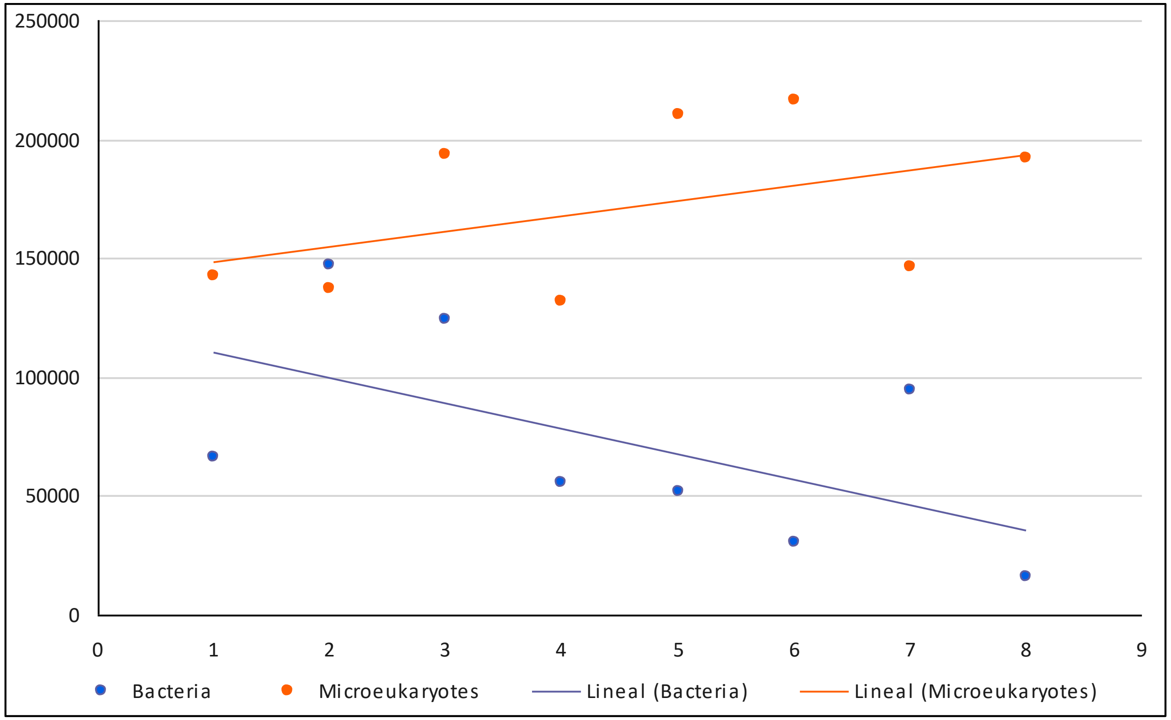

Figure S3. - Examples of human and animal-derived bacteria and microeukaryotes in the glacier samples. Graphic representation of the number of 16S rRNA and 18S rRNA sequences (in log) and their corresponding trend lines.

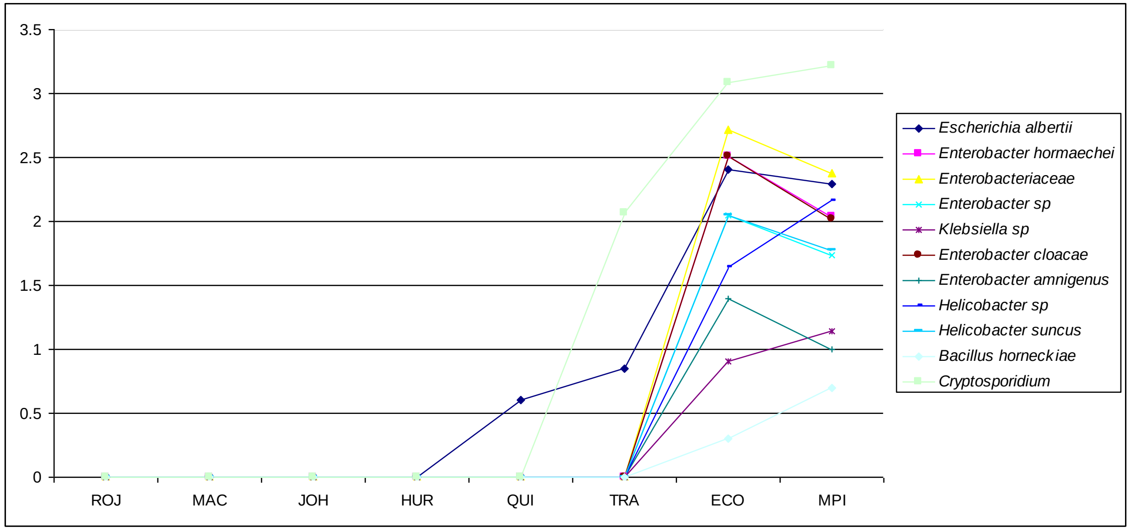

Figure S4. - The most abundant bacterial species participating in the sulfur cycle along the volcanic gradient. Graphic representation of the number of 16S rRNA sequences (in log) and their corresponding trend lines.

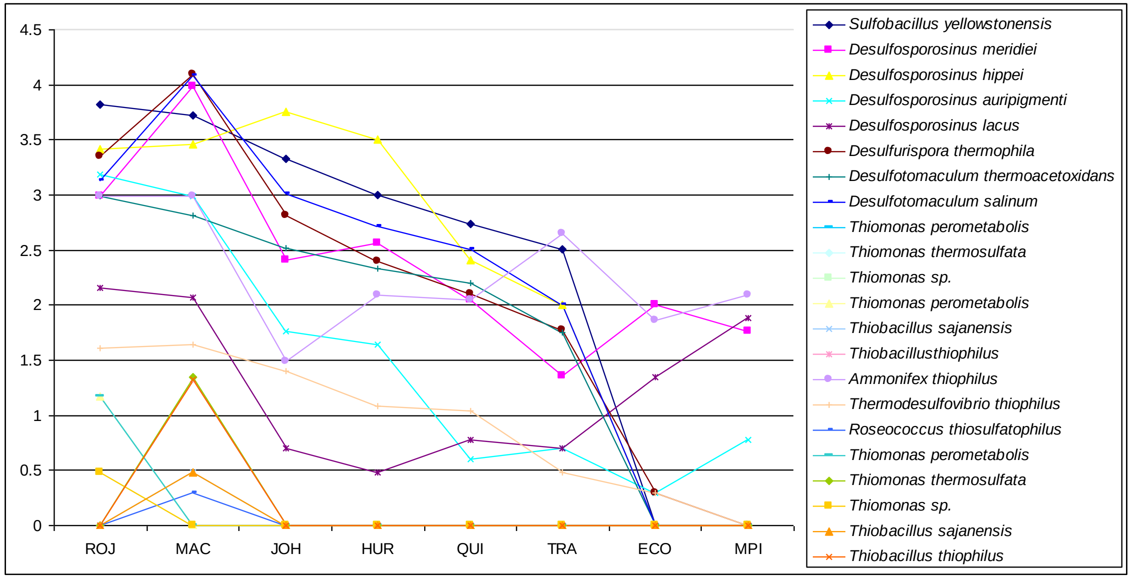

Supplement: Supplementary file 1 [file microorganisms-09-00392-s001.zip › microorganisms-1097587 supplementary/microorganisms-1097587-supplementary.pdf]
