# Supplementary material for: Microbial Community Structure Driven by a Volcanic Gradient in Glaciers of the Antarctic Archipelago South Shetland
Source: Microorganisms. 2021 Feb 14;9(2):392. doi: 10.3390/microorganisms9020392 (PMC7917679; doi:10.3390/microorganisms9020392)
Supplement: Supplementary file 1 [file microorganisms-09-00392-s001.zip › microorganisms-1097587 supplementary/microorganisms-1097587-supplementary.doc]

Supplementary Materials for:

Microbial community structure driven by a volcanic gradient in the Antarctic Archipelago South Shetland

*Eva García-Lopez, Sandra Serrano, Miguel Angel Calvo, Sonia Peña, Silvia Sanchez-Casanova Laura García-Descalzo and Cristina Cid**

* Corresponding Author: cidsc@inta.es

This file includes:

I. Supplementary Table: S1-S3

II. Supplementary Figures: S1-S4

**I. Supplementary Table**

**Table S1. Elemental concentrations in melt water.**

|  | **Deception** | | **Livingston** | | **Greenwich** | | **King George** | |
| --- | --- | --- | --- | --- | --- | --- | --- | --- |
|  | **ROJ** | **MAC** | **JOH** | **HUR** | **QUI** | **TRA** | **ECO** | **MPI** |
| **Ag** | BD^a^ | 0.002 (0.001) | 0.021 (0.001) | 0.088 (0.000) | 0.121 (0.001) | 0.084 (0.002) | 0.079 (0.003) | 0.065 (0.003) |
| **Al** | 0.627 (0.120) | 0.258 (0.004) | BD | BD | BD | BD | BD | BD |
| **As** | 0.226 (0.003) | 0.065 (0.004) | BD | BD | BD | 0.321 (0.004) | 0.338 (0.005) | 0.464 (0.022) |
| **B** | 13.499 (3.225) | 2.146 (1.700) | BD | 3.300 (0.630) | 5.528 (0.336) | 8.666 (1.336) | 12.345 (2.669) | 16.857 (2.700) |
| **Ba** | 5.060 (0.635) | 0.474 (0.021) | 0.633 (0.001) | BD | BD | BD | 2.042 (0.021) | 2.054 (0.033) |
| **Be** | 0.010 (0.001) | 0.013 (0.032) | 0.005 (0.001) | BD | 0.320 (0.004) | BD | 0.011 (0.004) | 0.088 (0.001) |
| **Bi** | BD | BD | 0.124 (0.007) | 0.211 (0.006) | 1.441 (0.004) | 0.369 (0.001) | 0.202 (0.021) | 0.008 (0.021) |
| **Br** | 7.740 (2.367) | 21.597 (7.254) | 10.741 (1.254) | 9.214 (2.369) | 50.247 (5.375) | 393.390 (45.778) | 465.088 (46.335) | 481.294 (10.478) |
| **C** | 4593.276 (214.778) | 4000.280 (879.225) | 3373.132 (214.478) | 1019.992 (52.144) | 1094.543 (50.214) | 2147.354 (588.214) | 3372.365 (244.321) | 3047.831 (354.877) |
| **Ca** | 1083.320 (20.789) | 547.369 (74.222) | BD | 99.654 (14.022) | BD | BD | 79.052 (11.225) | BD |
| **Cd** | 3.932 (0.077) | 7.116 (1.224) | BD | BD | BD | BD | BD | 0.066 (0.004) |
| **Cl** | 47406.605 (1.247) | BD | BD | BD | BD | 1.478 (0.240) | 113051.469 (1.225) | 118931.314 (14.225) |
| **Co** | BD | 0.073 (0.003) | 0.018 (0.002) | BD | 0.045 (0.004) | 0.030 (0.001) | 0.020 (0.000) | 0.012 (0.001) |
| **Cr** | 0.345 (0.002) | 0.211 (0.011) | BD | BD | BD | BD | BD | 0.093 (0.002) |
| **Cu** | 11.355 (0.522) | 11.869 (0.744) | 11.812 (0.211) | 10.258 (1.225) | 8.721 (1.336) | 9.354 (2.547) | 11.929 (3.214) | 13.348 (1.224) |
| **Fe** | 20.967 (3.254) | 15.369 (4.217) | 3.560 (0.544) | 10.369 (1.224) | BD | 1.330 (0.333) | BD | BD |
| **Ga** | BD | 0.004 (0.001) | BD | 0.010 (0.002) | 0.033 (0.001) | 0.014 (0.002) | BD | 0.004 (0.001) |
| **Ge** | BD | 0.003 (0.000) | BD | BD | BD | BD | BD | BD |
| **K** | 808.828 (50.226) | 310.048 (71.366) | 137.269 (33.666) | 125.698 (21.547) | 118.292 (32.144) | 54.369 (3.214) | 1833.946 (25.221) | 1680.072 (88.221) |
| **Li** | 3.327 (1.224) | 0.095 (0.012) | BD | BD | 0.214 (0.005) | 0.300 (0.012) | 0.308 (0.033) | 0.628 (0.022) |
| **Mg** | 5400.494 (33.211) | 3955.000 (65.214) | 28.408 (2.369) | 14.258 (3.655) | 207.091 (5.698) | 1354.159 (210.366) | 110.659 (23.364) | 2350.691 (562.314) |
| **Mn** | 1.563 (0.211) | 3.317 (0.214) | 1.160 (0.321) | 1.159 (0.333) | 1.158 (0.321) | 4.369 (0.986) | 6.504 (1.123) | 7.355 (1.225) |
| **Mo** | 0.051 (0.002) | 0.014 (0.003) | 0.154 (0.021) | 0.254 (0.021) | 0.583 (0.049) | 0.458 (0.055) | 0.328 (0.023) | 0.205 (0.008) |
| **Na** | 1173.431 (236.147) | 2269.103 (6.366) | 765.198 (63.321) | 700.154 (50.654) | 665.643 (20.354) | 874.321 (22.685) | 20269.703 (2147.369) | 18269.703 (3.254) |
| **Ni** | 35.357 (2.369) | 34.792 (5.487) | 26.801 (5.698) | 10.369 (6.321) | 0.231 (0.054) | 11.254 (1.566) | 26.716 (0.251) | 28.899 (0.999) |
| **P** | 1.878 (0.014) | 8.576 (1.123) | 14.981 (3.214) | BD | 37.028 (1.654) | BD | 124.052 (54.214) | 125.369 (12.365) |
| **Pb** | 0.050 (0.002) | 0.074 (0.005) | 0.009 (0.002) | BD | BD | 0.002 (0.001) | BD | BD |
| **Rb** | 0.260 (0.003) | 0.070 (0.006) | 0.059 (0.006) | 0.084 (0.008) | 0.102 (0.044) | 0.254 (0.055) | 0.479 (0.021) | 0.544 (0.005) |
| **S** | 16258.354 (1478.654) | 14509.753 (1456.325) | BD | BD | BD | 12698.000 (541.326) | 11748.304 (232.666) | 6733.097 (9.321) |
| **Sc** | BD | 0.015 (0.003) | BD | 0.123 (0.014) | 0.288 (0.044) | 0.111 (0.022) | BD | 0.022 (0.002) |
| **Se** | BD | BD | BD | BD | 1.525 (0.012) | 1.200 (0.055) | 1.012 (0.211) | BD |
| **Si** | 39.693 (1.254) | 30.654 (1.224) | BD | BD | BD | BD | BD | BD |
| **Sr** | 15.342 (1.369) | 1.044 (0.006) | 1.073 (0.006) | 2.365 (0.547) | 3.058 (0.598) | 10.258 (1.365) | 24.591 (2.323) | 26.740 (6.333) |
| **Ti** | 6.155 (1.254) | 1.389 (0.006) | BD | BD | BD | BD | BD | 7.084 (0.987) |
| **V** | 0.780 (0.005) | 0.100 (0.002) | 0.040 (0.001) | 0.090 (0.008) | 0.115 (0.025) | 0.222 (0.021) | 0.400 (0.020) | 0.569 (0.005) |
| **Y** | BD | BD | BD | BD | 0.060 (0.009) | 0.010 (0.002) | 0.016 (0.002) | 0.005 (0.001) |
| **Zn** | 62.056 (10.369) | 16.980 (2.156) | 10.932 (0.009) | 12.547 (1.354) | 15.201 (1987.321) | 20.147 (5.365) | 29.659 (2.366) | 35.105 (3.221) |
| **Zr** | BD | BD | 0.009 (0.003) | 0.054 (0.005) | 0.062 (0.055) | 0.024 (0.003) | 0.005 (0.001) | BD |

Values represent averages and s.d. (in parentheses) calculated from three sampling points from each study site; Elemental concentrations are in ppb; ^a^BD: below detection.

**Table S2. Analysis of bacterial 16S retrieved from ice samples.** OTU level aggregate counts of 3 sampling replicates.

File: Table S2.xlsx

**Table S3. Analysis of eukaryotic 18S rRNA retrieved from ice samples.** OTU level aggregate counts of 3 sampling replicates.

File: Table S3.xlsx

**Table S4. Number of sequences belonging to the most abundant OTU** in each sampling point

| **Glacier** | | **ROJ** | **MAC** | **JOH** | **HUR** | **QUI** | **TRA** | **ECO** | **MPI** |
| --- | --- | --- | --- | --- | --- | --- | --- | --- | --- |
| **Bacteria** | **OTU^a^** | *Sulfobacillus yellowstonensis* | *Polaromonas* | *Polaromonas* | *Pedobacter* | *Flavobacterium* | *Pedobacter* | *Clostridium frigoris* | *Sphingomonas* |
|  | **No. sequences^b^** | 6548 | 17501 | 69004 | 19897 | 13654 | 3332 | 38406 | 4348 |
| **Eukarya** | **OTU^a^** | *Thalassiosira antarctica* | *Hydrurus foetidus* | Glissomonadida | *Bodomorpha* sp. | *Cocconeis stauroneiformis* | *Urospora neglecta* | *Sporobolomyces roseus* | *Hyphozyma variabilis* |
|  | **No. sequences^b^** | 22915 | 64913 | 76908 | 49696 | 69106 | 80437 | 36098 | *33857* |

^a^Most abundant OTU in each glacier

^b^Number of total sequences from the most abundant OTU in each glacier

**II. Supplementary Figures**

**Figure S1.** - Rarefaction curves determined for 16S rRNA and 18S rRNA gene clones. Rarefaction curves indicate the observed OTUs at a genetic distance of 3%. (A) Bacteria. (B) Eukarya.

**Figure S2.** **-** Number of bacterial sequences and microeukaryote sequences along the volcanic gradient. Graphic representation of the number of 16S rRNA and 18S rRNA sequences and their corresponding trend lines.

**Figure S3.** **-** Examples of human and animal-derived bacteria and microeukaryotes in the glacier samples. Graphic representation of the number of 16S rRNA and 18S rRNA sequences (in log) and their corresponding trend lines.

**Figure S4.** **-** The most abundant bacterial species participating in the sulfur cycle along the volcanic gradient. Graphic representation of the number of 16S rRNA sequences (in log) and their corresponding trend lines.
